# A plasmid-based *lacZα* gene assay for DNA polymerase fidelity measurement

**DOI:** 10.1016/j.ab.2012.10.019

**Published:** 2013-02-15

**Authors:** Brian J. Keith, Stanislaw K. Jozwiakowski, Bernard A. Connolly

**Affiliations:** a Institute of Cell and Molecular Biosciences (ICaMB), Newcastle University, Newcastle upon Tyne, NE2 4HH, UK; b MRC Genome Damage and Stability Centre, University of Sussex, Brighton, BN1 9RQ, UK

**Keywords:** DNA polymerase, Polymerase fidelity, *lacZ*α reporter gene

## Abstract

A significantly improved DNA polymerase fidelity assay, based on a gapped plasmid containing the *lacZα* reporter gene in a single-stranded region, is described. Nicking at two sites flanking *lacZα,* and removing the excised strand by thermocycling in the presence of complementary competitor DNA, is used to generate the gap. Simple methods are presented for preparing the single-stranded competitor. The gapped plasmid can be purified, in high amounts and in a very pure state, using benzoylated–naphthoylated DEAE–cellulose, resulting in a low background mutation frequency (∼1 × 10^−4^). Two key parameters, the number of detectable sites and the expression frequency, necessary for measuring polymerase error rates have been determined. DNA polymerase fidelity is measured by gap filling in vitro, followed by transformation into *Escherichia coli* and scoring of blue/white colonies and converting the ratio to error rate. Several DNA polymerases have been used to fully validate this straightforward and highly sensitive system.

DNA replication is a complex process that requires a multiprotein assembly (the replisome) to bring about the efficient, rapid, and accurate copying of the genome [1,2]. The replisome minimizes the appearance of detrimental mutations, thereby maintaining genomic stability, and the base substitution error rate of the *Escherichia coli* replication machinery in vivo is approximately 1 × 10^−7^ to 10^−8^ [3]. DNA polymerases make a major contribution to accuracy, and some replicative polymerases result in only one error per 10^5^/10^6^ bases incorporated, although other enzymes (e.g., those involved in translesion bypass) are less accurate [4,5]. DNA polymerases are essential components of many biotechnological applications, especially the polymerase chain reaction (PCR),^1^ where accuracy is important [6–8]. Therefore, it is important both to understand the mechanisms that contribute to DNA polymerase fidelity and to develop assays sensitive enough to detect the infrequent errors that many polymerases make.

Several approaches to measure fidelity have been described; however, many are complicated or do not detect the whole spectra of mutations that may occur [9–11]. One of the most successful methods uses replication of a gap in the bacteriophage M13mp2 *lacZα* gene, encoding an inactive fragment of β-galactosidase, the α-peptide. When accurately copied, and subsequently introduced into *E. coli* that bears a complementing copy of the remaining β-galactosidase gene, functional β-galactosidase is reconstituted, resulting in the hydrolysis of X-gal and blue bacterial plaques [12]. Inaccurate polymerase activity may result in a defective α-peptide, eventually resulting in reduced or abolished β-galactosidase activity, indicated by light blue or colorless plaques. The error rate is calculated from the blue/colorless plaque ratio, and further information can be obtained from DNA sequencing. This method allows the detection of all 12 possible base substitutions as well as insertions and deletions in varying sequence contexts, is very well characterized, and has been used extensively by the Kunkel group for many investigations of DNA polymerases [4,5,12,13].

Previously, we described a plasmid-based method for measuring polymerase fidelity using pSJ1, a derivative of pUC18 [14]. In pSJ1, a segment encoding *lacZα* is flanked by two single-strand nicking endonuclease sites, allowing one of the DNA duplex strands to be specifically cut on both sides of the *lacZα* gene. Removal of the nicked DNA fragment results in a gapped plasmid containing the *lacZα* gene in the single-stranded region. The accuracy of a polymerase can be determined by copying the gene in vitro and then introducing the plasmid into *E. coli,* an approach similar in concept to that described above for the bacteriophage system. The plasmid-based system benefits from the simple preparation of pSJ1 in reasonable quantities and straightforward use in fidelity evaluation [14]. Because plasmids are compatible with many cell types, gapped variants can be used to study DNA replication and repair in vivo [15]. Nevertheless, the pSJ1 method is currently underdeveloped as compared with the much longer established bacteriophage approach. In particular, two key parameters, the number of detectable mutations (alterations in *lacZα* that result in an inactive gene and a white phenotype) and the expression frequency (the degree to which the polymerase-synthesized strand is expressed in *E. coli*), have yet to be determined. Knowledge of these features is required for converting the observed ratio of blue/white colonies/plaques to a polymerase error rate, measured as mistakes per base incorporated [12,13]. In this study, both the detectable mutations and the expression frequency have been measured, making the plasmid system fully equivalent to the bacteriophage. Furthermore, considerable improvements in the preparation and purification of the gapped plasmids are outlined, resulting in larger amounts of purer product and lowered background mutation rates. Together, these refinements substantially increase the scope and power of plasmid methods for polymerase fidelity measurements.

## Materials and methods

### Materials

Deoxynucleotide triphosphates (dNTPs), lambda (λ)-exonuclease, ExoIII, DNA ligase, and the restriction endonucleases EcoRI, DpnI, NdeI, and SalI were supplied by Fermentas (York, UK). Taq-Pol, for use in routine PCR applications, was also purchased from Fermentas. The nicking enzymes Nt.Bpu10I and Nt.BbvCI and the restriction enzyme BbvCI were purchased from New England Biolabs (Ipswich, MA, USA). Plasmid-safe DNase was supplied by Cambio (Cambridge, UK). PCR cleanup, nucleotide removal, and miniprep and maxiprep kits were obtained from Qiagen (Crawley, UK). Benzoylated–naphthoylated DEAE–cellulose (BND–cellulose) was obtained from Sigma–Aldrich (Gillingham, UK). Top10 competent *E. coli* was obtained from Invitrogen (Paisley, UK), and Velocity DNA polymerase was obtained from Bioline (London, UK). *Pyrococcus furiosus* family B DNA polymerase (Pfu-Pol B), Pfu-Pol B exo^–^ (D141A/E143A) and Pfu-Pol B exo^–^ error prone (D141A/E143A/D473G), was purified as described previously [16,17]. A Taq-Pol overexpressing system was created by inserting the Taq-Pol gene (amplified by PCR from *Thermus aquaticus* genomic DNA) between the NdeI and SalI restriction endonuclease sites of pET-17b (Novagen, Merck–Millipore, Watford, UK) using DNA ligase. The resulting pET-17b (Taq-Pol) was used to transform *E. coli* BL21(DE3) (pLysS) and the polymerase purified exactly as described for Pfu-Pol [16,17]. ImageQuant colony-counting software was provided by GE Healthcare (Hatfield, UK).

### Preparation of pSJ2 and pSJ3

Two plasmids pSJ2 and pSJ3, based on pUC18 and M13mp2, were produced for DNA polymerase fidelity assays. pSJ2 was prepared and supplied by Biomatik (Wilmington, DE, USA). pSJ3 was assembled in-house commencing from pUC18. A PCR-based site-directed mutagenesis protocol [18] was used to (i) flank the *lacZα* gene with N(t/b)Bpu10I nicking sites (equivalent to a Bpu10I restriction site) and (ii) delete the dam methylase (GATC) site within this gene in order to avoid cellular mismatch-directed repair mechanisms [19]. Reactions were performed in a 100-μl reaction volume and consisted of 200 ng of template DNA, 1.5 μM of each primer, 400 μM of each dNTP, 4 U of Velocity, and the supplier’s recommended buffer. The reaction was subjected to 32 PCR cycles of 35 s at 95 °C, 35 s at 55 °C, and 3 min 30 s at 70 °C. An initial denaturing step of 98 °C for 1 min and a final 10-min elongation step were also performed. The polylinker in pUC18 was deleted using the PCR-based SLIM (site-directed ligase independent mutagenesis) approach with appropriately designed primers [20]. Two individual reactions, each half the volume described above, were submitted to 22 PCR cycles. These reactions were then combined to form a new reaction and subjected to a further 22 PCR cycles after the addition of a further 2 U of Velocity. Each PCR used the same initial, extension, and final cycles described above. After all mutagenesis PCRs, the resulting plasmid was purified using a PCR cleanup kit according to the supplier’s protocol. With the product eluted in 35 μl of H_2_O preheated to 50 °C. 15 μl of this product was supplemented with 7 U of DpnI in the supplier’s recommended buffer and incubated at 37 °C for 3 h to degrade the parental plasmid. Then 1 μl of this reaction was used to transform 60 μl of competent *E. coli* Top 10 cells, and recovered plasmids were completely sequenced to confirm the integrity of the mutagenesis.

### Identifying detectable sites in pSJ2 and pSJ3

The *lacZα* DNA sequences in both pSJ2 and pSJ3 are very similar to that found in M13mp2. Therefore, nearly all of the detectable sites (i.e., base changes that result in an inactive α-peptide) are known. The status of the small number of *lacZα* bases in pSJ2 and pSJ3, not found in the bacteriophage, was determined using site-directed mutagenesis. The deoxynucleoside at each of these uncharacterized positions was separately changed to all other three bases (for the promoter region) and to all bases that resulted in a new amino acid (for the protein coding region). PCR-based site-directed mutagenesis [18] was used, as described above, to make these alterations.

### Preparation of single-stranded *lacZα* competitor DNA

Single-stranded DNA was generated using a PCR/λ-exonuclease method [14]. Two primers, one containing a 5′-phosphate and the second containing three phosphorothioates at the phosphodiesters nearest the 5′ end (see Fig. S1 in Supplementary material), were used to amplify the *lacZα* gene in pSJ2 and pSJ3. The PCR mixture (100 μl) consisted of: plasmid (300 ng), primers (1.5 μM of each), dNTPs (200 μM of each), MgCl_2_ (1.5 mM), and Taq-Pol (5 U as defined by the supplier) in the supplier’s recommended buffer. PCR consisted of 30 cycles: 35 s at 95 °C (1 min at 98 °C for the first cycle), 35 s at 55 °C, and 30 s (90 s on last cycle) at 70 °C. The amplified *lacZα* DNA was purified with a PCR cleanup kit. The 5′-phosphorylated strand was then specifically degraded by treating 2  μg of amplified product with 5 U of λ-exonuclease in 50 μl of the supplier’s recommended buffer for 30 min at 37 °C. The resulting single-stranded DNA was purified using a nucleotide removal kit. Alternatively, chemically synthesized oligodeoxynucleotides were used as competitors (Fig. S1).

### Preparation of gapped DNA

Plasmid (40 μg) was digested with 40 U of Nt.BbvCI (pSJ2) or Nt.Bpu10I (pSJ3), both enzymes cut twice on the coding strand flanking the *lacZα* gene. Nicking was performed for 3 h at 37 °C in 1 ml of the supplier’s recommended buffer. This reaction was carried out five times in parallel (total of 200 μg plasmid). Nicked plasmid was gapped in 48 simultaneous reactions, each containing 3.5 μg (∼2 pmol) of plasmid using either a 10-fold molar excess of PCR/λ-exonuclease-prepared single-stranded competitor (for pSJ2) or a 50-fold molar excess of chemically synthesized oligodeoxynucleotide competitor (for pSJ3). The aliquots of nicked plasmid and competitor DNA were each incubated in 100 μl of the nicking buffer and subjected to three heat/cool cycles of 95 °C for 1 min, 60 °C for 10 min, and 37 °C for 20 min. The reaction mixtures were then pooled, and gapped plasmid was separated from excess competitor and any displaced double-stranded *lacZα* DNA using four passes (reducing the volume ∼ 10-fold in each pass) through a 100-kDa cutoff Amicon ultrafilter (Millipore). During ultrafiltration, the gapped plasmid was simultaneously equilibrated to TE buffer (10 mM Tris–HCl [pH 8.0] and 1 mM EDTA [ethylenediaminetetraacetic acid]). The process yielded 160 μg of crude gapped plasmid, which was subsequently purified using 2 g of BND–cellulose prepared as a 50% suspension in TE buffer containing 1 M NaCl. The DNA was gently agitated with 4 ml of BND–cellulose slurry for 10 min at room temperature, and the mixture was loaded into a column. The resin was washed with 60 ml of TE buffer containing 1.0 M NaCl, and the gapped plasmid was eluted with CFS buffer (TE buffer containing 1 M NaCl, 2% [w/v] caffeine, and 50% [v/v] formamide). Then 1-ml fractions were collected and assayed for the presence of gapped plasmid using 1% agarose gel electrophoresis. Appropriate fractions were pooled, and the gapped plasmid was reequilibrated to TE buffer using ultrafiltration as described above. Purified gapped plasmid (80 μg) was stored frozen in 20-μl aliquots at −20 °C.

### Expression frequency determination

This investigation was carried out only with pSJ2. Initially, PCR-based site-directed mutagenesis was used to destroy the upstream nicking site, giving a derivative (pSJ2A) with only a single downstream nicking site. A further round of mutagenesis with pSJ2A was used to make a solitary base change that resulted in an in-phase premature stop codon toward the beginning of the *lacZα* coding sequence, yielding pSJ2B. The two plasmids, pSJ2A and pSJ2B, were then used to produce a heteroduplex with a single-base mismatch [15]. To achieve this, pSJ2A (40 μg) was nicked on one strand at its single Nt.BbvCI site (40 U of enzyme for 16 h at 37 °C) and the cut strand was degraded with ExoIII (2400 U of ExoIII for 20 min at 37 °C in 1 ml of the supplier’s recommended buffer). The resulting single-stranded circular DNA was purified using a PCR cleanup kit. pSJ2B (10 μg) was cut on both strands at its single BbvCI site by digestion with 40 U of the restriction endonuclease BbvCI (16 h at 37 °C), and the resulting linear duplex was purified with a PCR cleanup kit. The cutting/purification cycle with pSJ2B was repeated twice to ensure complete digestion. The single-stranded circular DNA (derived from pSJ2A) and linear duplex (derived from pSJ2B) were mixed in a 1:1.5 M ratio, heated to 95 °C, and cooled slowly to room temperature to produce the required circular heteroduplex DNA containing a single nick. The resulting solution was treated with plasmid-safe DNase, which destroys any remaining single-stranded circular DNA and linear duplex DNA but does not degrade the heteroduplex. Plasmid-safe DNase (10 U) was used in the supplier’s recommended buffer supplemented with 1 mM ATP. After treatment for 15 h at 37 °C, the heteroduplex was purified with a PCR cleanup kit. As above, the DNaseI/purification cycle was repeated twice more to maximize degradation of unwanted starting components. To produce an appropriate control, this process was repeated, but both the single-stranded circular DNA and the linear duplex were derived from pSJ2A, ultimately resulting in a wild-type functional *lacZα* gene. The heteroduplex and the control were used to transform *E. coli* Top10 (see below), and the ratio of blue/white colonies was determined, allowing calculation of the expression frequency.

### Background mutation rate determination

The background mutation frequency was determined by transforming 60 μl of *E. coli* Top 10 cells [14] with 1 μl of the plasmid under investigation (pSJ2, pSJ3, and their gapped derivatives). Following transformation, these cells were then plated on LB agar supplemented with 40 μg/ml X-gal, 100 μg/ml ampicillin, and 250 μM IPTG (isopropyl β-d-1-thiogalactopyranoside). These plates were incubated at 37 °C for 16 h and then scored for blue and white colonies.

### DNA polymerase fidelity assay

The gapped derivatives of pSJ2 and pSJ3 were filled by the DNA polymerase under investigation using 20 μl of 20 mM Tris–HCl (pH 8.0), 10 mM KCl, 2 mM MgSO_4_, 10 mM (NH_4_)_2_SO_4_, 0.1% Triton X-100, 2 μg of bovine serum albumin, and 250 μM of each dNTP. The concentrations of the DNA polymerase and the gapped plasmid were 100 and 1 nM, respectively. Extension reactions were carried out at 70 °C for 30 min and, after this time, were examined using 1% agarose gel electrophoresis. Approximately 9 μl of the reaction mixture was analyzed directly by electrophoresis, and a further 9 μl was tested following digestion with 5 U of EcoRI (30 min at 37 °C). Provided that the electrophoresis/EcoRI analyses indicated complete extension, 1 μl of the remaining original reaction mixture was used for the transformation of competent *E. coli* Top 10 cells (60 μl). Transformed cells were plated on LB agar and scored for blue/white colonies as described above. To determine the appropriate background mutation rate for polymerase extension assays, the protocol was carried out in the absence of DNA polymerase (in this case, electrophoretic analysis is not required).

### Colony counting

To facilitate counting of large numbers of blue and white *E. coli* colonies, a digital camera was used to take images of the plates, which were then analyzed by ImageQuant colony-counting software.

### DNA sequencing

Mutant (white) colonies were grown overnight at 37 °C in LB medium containing 100 μg/ml ampicillin. Plasmid was purified using miniprep kits and sent to be sequenced. (GATC Biotech, Cambridge, UK).

## Results

### Redesigning the *lacZα* gene to give pSJ2 and pSJ3

**Fig.1:**
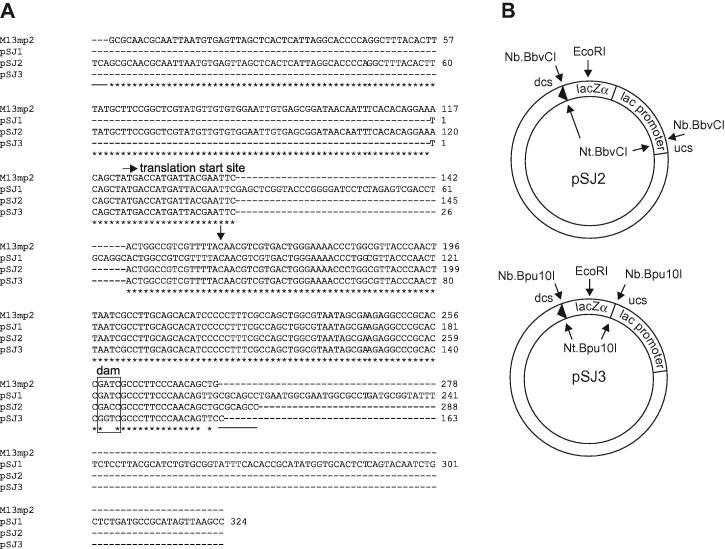
The main features of pSJ2 and pSJ3, plasmids useful for measuring DNA polymerase fidelity. (A) The lacZα peptide and *lac* promoter sequences of M13mp2, pSJ1, pSJ2, and pSJ3. Only the bases actually used in fidelity determination are shown; thus, pSJ1 and pSJ3 contain the *lac* promoter, but because of the nicking site positions, this element is not used in fidelity assays with these two plasmids. The symbol ∗ indicates identical bases in the lacZα peptide (for all four sequences) and in the *lac* promoter (for M13mp2 and pSJ2). The underlined sequences are the extra bases at the extremities of pSJ2, necessary for introducing the nicking sites. The ATG triplet that encodes the first methionine in the lacZα peptide is shown, as is the dam methylase (GATC) site altered in pSJ2 and pSJ3. The C shown with the symbol ↓ is the base altered in pSJ2 to introduce a stop codon for expression frequency determination. In all cases, the coding sequence of *lacZα* is given, which corresponds to the bases on the inner circle of the plasmids illustrated. (B) Plasmid maps of pSJ2 and pSJ3 showing the location and orientation of the *lacZα* sequences and the nicking endonuclease sites, N(t/b)BbvCI for pSJ2 and N(t/b)Bpu10I for pSJ3. The EcoRI site used for analytical purposes is also shown. The abbreviations dcs and ucs stand for downstream and upstream cutting sites, respectively.

To rigorously evaluate the error rate of a polymerase as mistakes made per base incorporated, it is necessary to know the number of detectable mutations in the indicator gene. A particular advantage of the bacteriophage system, based on the *lacZα* sequence present in M13mp2, is that a large number of experiments have characterized nearly every detectable mutation, that is, exactly which base substitutions and insertions/deletions result in an inactive α-peptide and, hence, a change in plaque color from blue to white [4,5,12,13,21,22]. The plasmid previously used, pSJ1, encodes an active α-peptide, but its gene sequence is derived from pUC18 and, ultimately, M13mp18 [14]. Thus, as shown in Fig. 1A, the M13mp2 and pSJ1 *lacZα* sequences vary somewhat, making it complicated to apply the wealth of knowledge available for the phage system to this plasmid. As a remedy, two new plasmids have been designed with *lacZα* sequences that more closely match that found in M13mp2. One of the plasmids, pSJ2, has a *lacZα* sequence nearly identical to M13mp2, mainly differing in the presence of three and seven extra bases at the 5′ and 3′ ends, respectively (underlined in Fig. 1A), necessary for introducing the N(t/b).BbvCI nicking sites (Fig. 1B). The second plasmid, pSJ3, matches M13mp2 closely over the *lacZα* sequence that actually encodes protein (the additional upstream sequences present in both M13mp2 and pSJ2 are predominantly the *lac* promoter), with minor base changes at the 5′ and 3′ termini to accommodate the N(t/b).Bpu10I nicking enzyme used in this case (Fig. 1B). Further very slight sequence deviations are caused by the removal of dam methylation sites (GATC) from both pSJ2 and pSJ3 (Fig. 1A) to eliminate any complications arising from dam-targeted base mismatch repair [19]. With pSJ2 and pSJ3, the sizes of the gapped regions ultimately used for DNA polymerase fidelity assay are 288 and 163 bases, respectively.

### Detectable sites in pSJ2 and pSJ3

The main features of pSJ2 and pSJ3 are illustrated in Fig. 1B. Both plasmids contain a *lacZα* gene sequence (given in full in Fig. 1A) flanked by two nicking endonuclease sites. The nicking enzymes (N.BbvCI and N.Bpu10I) are available as “t” or “b” forms, which specifically cut the “inner” strand (containing the *lacZα* coding sequence) or the “outer” strand (containing the *lacZα* noncoding sequence) of the plasmids shown in Fig. 1B. Thus, the alternative use of “t” and “b” gives two orientations of the gapped plasmid, named “+” (inner coding strand removed) and “−” (outer noncoding strand removed) in earlier studies with pSJ1 [14]. All of the studies reported in this article used the “+” gapped forms, that is, had the inner coding strand removed with the “t” variants of the nicking enzymes. However, a small number of investigations with the “−” orientations gave identical results (data not shown). A unique EcoRI restriction site is located within the *lacZα* gene, important for subsequently analyzing the efficiency of the gapping process and for monitoring extension of the gapped plasmid by DNA polymerase. Because pSJ2 and pSJ3 have nearly identical *lacZα* sequences to M13mp2, nearly all of the detectable sites (i.e., changes to bases that result in an inactive α-peptide) are known by simple comparison. The detectability of the few *lacZα* bases in pSJ2 and pSJ3, absent in the bacteriophage, was determined using site-directed mutagenesis. Each novel base was systematically changed to all others (in the case of the *lac* promoter) or, with protein coding sequences, to any base that resulted in a change of amino acid. Only one new detectable site, for base substitutions, was found. With the underlined CGCAGCC sequence in pSJ2 (Fig. 1B), an A to C change gave an inactive lacZα peptide and white colonies. All other substitutions were silent. The seven extra bases at the end of pSJ2 gave detectable insertions and deletions, although the three at the beginning were silent. The numbers of detectable sites for M13mp2, pSJ2, and pSJ3 are summarized in Table 1 and are similar, as expected, provided that the smaller size of the *lacZα* gene in pSJ3 is taken into account. A full list of the detectable sites is given in the Supplementary material (Fig. S2).

### Preparation of gapped pSJ2 and pSJ3

**Fig.2:**
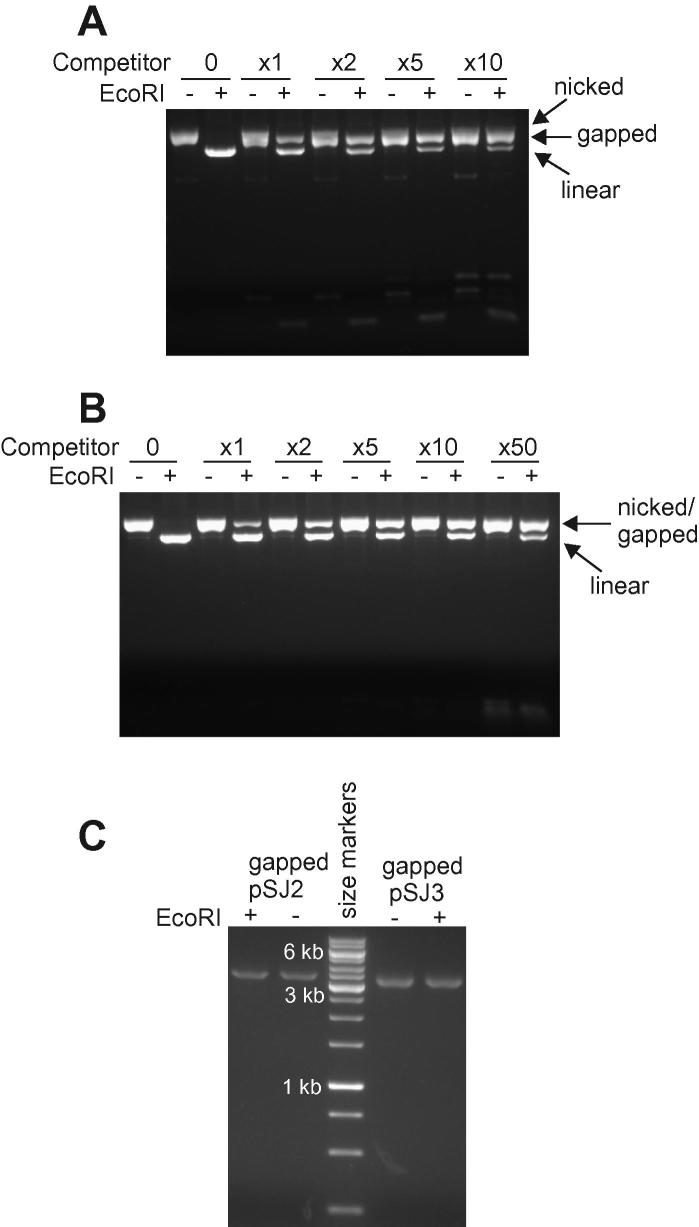
Preparation of gapped pSJ2 and pSJ3. (A) Gel electrophoretic analysis of the generation of gapped pSJ2. The nicked form (previously prepared by reaction of pSJ2 with Nt.BbvCI) is converted to the desired gapped product by heat/cool cycles with a 288-base competitor oligodeoxynucleotide (excess used indicated above the gel lanes). The nicked starting material and the gapped product are poorly resolved. However, EcoRI converts the nicked plasmid (but not the gapped plasmid) to the well-separated linear form, enabling analysis of the progress of the gapping reactions. (B) Gel electrophoretic analysis of the preparation of gapped pSJ3. The nicked form (previously prepared by reaction of pSJ3 with Nt.Bpu10I) can be converted to the desired gapped product using heat/cool cycles with two 80-base competitors (excess used indicated above the gel lanes). The starting nicked plasmid and the gapped product are not resolved, and EcoRI digestion, which converts remaining nicked pSJ3 to the linear form but does not act on the gapped form, is required for analysis. (C) Gel electrophoresis of pSJ2 and pSJ3 following purification using BND–cellulose. Analyses were carried out with or without pretreatment of EcoRI to fully control for any contaminating nicked plasmid. The size marker is a GeneRuler 1-kb ladder (Fermentas), with the three intense bands being 1-, 3-, and 6-kb products, as indicated on the gel.

With pSJ1, preparation of the gapped derivative required cutting the plasmid with the appropriate nicking enzyme and heating in the presence of a single-stranded competitor DNA [14]. The competitor was designed to be complementary to the nicked strand and so to sequester it during the heat/cool cycle to give the gapped plasmid. Previously, single-stranded competitor preparation used PCR amplification with two primers: one with a 5′-OH and the other containing a 5′-phosphate. Subsequent degradation of the PCR product with λ-exonuclease, an enzyme that preferentially degrades duplex DNA from ends containing a 5′-phosphate, yields the desired single strand [23]. Unfortunately, λ-exonuclease does not absolutely discriminate between 5′-phosphorylated and 5′-OH ends; therefore, some destruction of the required strand occurs. To further improve selectivity, the 5′-phosphorylated PCR primer has been combined with one containing a 5′-OH group and phosphorothioate modifications at the three internucleotide phosphodiester groups nearest the 5′ end (Fig. S1). DNA substituted with phosphorothioates is commonly observed to be nuclease resistant [24], and the new combination of primers is expected to better target λ-exonuclease to the 5′-phosphorylated strand. Indeed, the new combination of primers reliably produced higher concentrations of competitor DNA than those seen when the phosphorothioates were missing. Typically, approximately 17.5 μg of single-stranded product was produced from 100-μl PCR volumes using the triple phosphorothioated primer. In contrast, approximately 7.5 μg of product was produced with a normal primer lacking phosphorothioate protection. A single-stranded competitor produced in this manner, 288 bases in length and fully complementary to the excised *lacZ* sequence, could be used to produce gapped pSJ2, as shown in Fig. 2A. In this figure, the starting nicked plasmid and desired gapped product are poorly resolved. However, treatment with the restriction endonuclease EcoRI converts the nicked plasmid (which contains a GAATTC EcoRI site in double-stranded DNA) to the linear form. The gapped plasmid, in which GAATTC is in single-stranded DNA, is inert toward EcoRI. The gapped and linear plasmids are well resolved (Fig. 2A), and the relative intensities of their bands are indicative of the gapping yield. In agreement with earlier studies using pSJ1, a 10-fold excess of the competitor was optimal [14].

Although the use of phosphorothioate-containing primers improves the PCR method for producing competitor, it is still difficult to generate large amounts of material, which is important because a 10-fold excess is required. As an alternative, the use of chemically synthesized DNA competitors has been explored. Standard solid phase synthesis of oligodeoxynucleotides is limited to lengths of approximately 100 bases [25], shorter than the gapped regions produced when using pSJ2 (288 bases) and pSJ3 (163 bases). However, two competitors approximately 80 bases in length, designed to almost completely cover the 163 nucleotide *lacZα* gene of pSJ3 (Fig. S1), were effective in gapping (Fig. 2B). Again EcoRI treatment was required to distinguish the starting nicked plasmid and the gapped product. These two oligodeoxynucleotides could be used without purification, but a high ratio of competitor to plasmid, 50:1, was needed (with the PCR/λ-exonuclease method, a 10-fold excess suffices). Nevertheless, the higher amounts of material available from chemical synthesis compared with PCR make this approach worthwhile. Unfortunately, the use of chemically synthesized DNA was relatively inefficient with pSJ2. Attempts to gap the 288-nucleotide *lacZα* region with two oligodeoxynucleotide sets (the first consisting of five strands and the second consisting of four strands, with each DNA strand between 50 and 80 bases long) were largely unsuccessful (Fig. S1). Much of the starting nicked double-stranded plasmid remained in the mixture, and little of the required gapped derivative was produced (see Fig. S3 in Supplementary material). Therefore, the PCR method for competitor preparation needed to be used with this plasmid.

### Purification of gapped pSJ2 and pSJ3

In earlier work, gapped pSJ1 was purified by gel electrophoresis, a method that limits the amount of product that can easily be prepared [14]. Gapped plasmids contain a stretch of single-stranded DNA, which results in the exposure of relatively hydrophobic bases, normally buried in double-stranded DNA. This enabled their isolation using BND–cellulose, a material that interacts strongly with hydrophobic regions and so binds tightly to gapped plasmids [26,27]. Elution of the gapped plasmids from BND–cellulose uses a solution of caffeine. Purification using BND–cellulose is simpler and quicker than gel electrophoresis and produces larger quantities of gapped plasmid. Starting from 200 μg of pSJ2 or pSJ3, 80 μg of gapped plasmid (40% yield) was typically obtained and shown by gel electrophoresis to be extremely pure (Fig. 2C). Treatment of the purified gapped plasmids with EcoRI did not give rise to an additional linear product of faster mobility, as would be expected in cases of contamination with nicked starting plasmid (Fig. 2C). The BND–cellulose purification can be scaled up to handle milligram (mg) quantities of plasmid. As described below, plasmids isolated using BND–cellulose resulted in a lower background mutation rate than that observed using electrophoresis, presumably arising from less damage to the DNA bases. This is a significant advantage for fidelity measurements with highly accurate DNA polymerases.

### Expression frequency of pSJ2 and pSJ3

When a polymerase makes a mistake during the filling in of a gapped plasmid, either a heteroduplex with a single-base mismatch or a frame shift usually results. However, this will be scored as a polymerase error only if the coding information in the newly synthesized strand is used to direct lacZα peptide synthesis. The gap-filled pSJ2 and pSJ3 plasmids used for *E. coli* transformation are dam-neutral; that is, all of the GATC sites outside the *lacZα* gene are fully methylated, and the gene itself lacks dam sequences. Therefore, hemi-methylated dam sequences, which would arise from polymerase filling of a *lacZα* gene containing GATC runs, are lacking. Hemi-methylation of dam sequences is used to guide mismatch repair in *E. coli* [19], and in its absence one would predict that base mismatches would be restored in an undirected manner. Thus, when a mismatch is produced, the polymerase introduced base and the original template base are each replaced 50% of the time, a number that corresponds to the expression frequency. However, polymerase-filled pSJ2 and pSJ3 also contain a nick at the 3′ end of the *lacZα* gene, which could influence mismatch repair [19], making it important to experimentally determine the expression frequency. To do this, a plasmid identical to that produced after a mistake in gap filling (i.e., with a detectable error in the equivalent of polymerase-generated strand and a 3′ nick) has been generated. Full details are given in the Supplementary material (Fig. S4). In brief, they involve changing a single C to a T (Fig. 1A) and converting a CAA codon (which specifies Gln13 in the lacZα peptide) to a TAA stop codon, giving a truncated inactive α-peptide. The sequences specifying an “active” and “inactive” lacZα peptide can be blended to give a heteroduplex with a G:T mismatch (Fig. S4). In this heteroduplex, the noncoding strand bears the wild-type triplet (TTG, specifying CAA on the coding strand following replication), and the coding strand possesses the stop codon (TAA). The coding strand also has a nick at the end of the *lacZα* gene; therefore, the coding and noncoding strands are equivalent to newly synthesized and parental sequences in a polymerase extension assay. Depending on how the G:T mismatch is repaired, either an active (blue colonies) or inactive (white colonies) lacZα peptide will be produced, with the ratio of blue/white colonies reporting on which base in the mismatch is changed. When the G:T heteroduplex was used to transform *E. coli,* 44.4% of the colonies observed were white, resulting from a truncation in the lacZα peptide due to the stop codon (Table 2). For completely random repair, a value of 50% would be expected; therefore, the value of 44.4% indicates that repair of base mismatches in pSJ2 (and presumably pSJ3) is essentially random. For all subsequent experiments, 0.444 has been used as the expression frequency. A control experiment used a homoduplex having a wild-type CAA (Gln13) codon but produced in exactly the same manner as the heteroduplex (Fig. S4). Transformation of *E. coli* with this control plasmid resulted in only blue colonies; no white colonies, which would indicate a mutation in the *lacZα* gene, were observed (Table 2).

### Background mutation frequency found with pSJ2 and pSJ3

Ideally, when a plasmid or bacteriophage containing the *lacZα* gene is used to transform a complementing *E. coli* host, all colonies/plaques should be blue in the presence of X-gal due to the production of an active β-galactosidase. Invariably, a small number of white colonies are seen, arising from damage to the bases (some of which will inactivate the α-peptide) during the manipulations necessary to prepare and purify the gapped substrate. The resulting background mutation frequency makes it difficult to investigate highly accurate polymerases, which often produce only few additional errors [12,22]. The background rates seen with BND–cellulose purified gapped pSJ2 and pSJ3 are summarized in Table 3 along with that found for starting pSJ2 itself. Very few white colonies are observed, making it difficult to determine the extremely low background mutation rate accurately. For comparison, the background seen using gapped pSJ2 purified by gel electrophoresis is also given, as are previous values observed for nicked pSJ1 [14] and M13mp2 [12,13]. It is clear that gapped pSJ2 purified using the BND–cellulose method shows a 5-fold reduction in background errors when compared with the gel extraction method. BND–cellulose purified gapped pSJ3 has an even lower background mutation frequency, almost certainly due to the shorter gap in *lacZα.* The very low backgrounds observed with pSJ2 and pSJ3 represent a significant improvement over earlier *lacZα*-based systems. Although only four white colonies were observed with pSJ2 and pSJ3, these have been fully sequenced to confirm the presence of a mutation. In three cases, a C → T/G → A transition was observed (Table 3), a mutation also overrepresented in an earlier study using pSJ1 [14]. This transition is most likely caused by the deamination of cytosine to uracil, a thymidine mimic.

### Validation of pSJ2 and pSJ3

**Fig.3:**
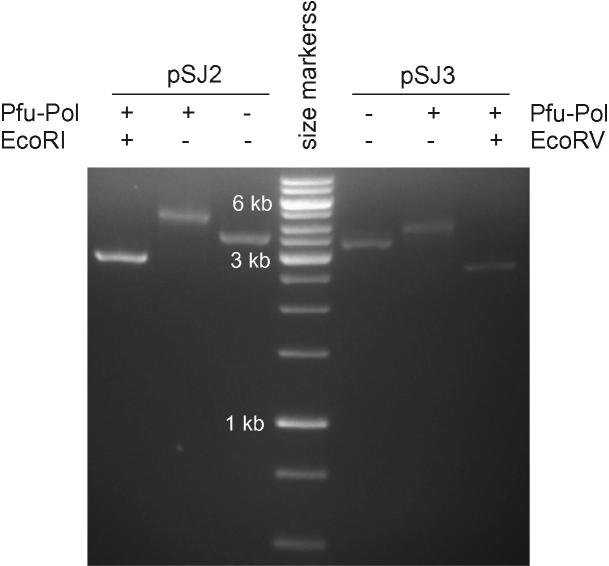
Filling in of gapped pSJ2 and pSJ3 with Pfu-Pol. The gel shows the starting gapped pSJ2 and pSJ3 (Pfu-Pol−, EcoRI−) and the extended product produced with the addition of Pfu-Pol (Pfu-Pol+, EcoRI−). A subsequent digestion with EcoRI (Pfu-Pol+, EcoRI+) converts the filled plasmid to the linear form, confirming extension by Pfu-Pol, which results in the EcoRI site becoming double-stranded. The size marker is a GeneRuler 1-kb ladder (Fermentas), with the three intense bands being 1, 3, and 6 kb in size, as indicated on the gel.

To check whether the new plasmids are suitable for assessing the fidelity of DNA polymerases, error rates have been determined for a number of well-characterized enzymes. Studies have been carried out with the Klenow fragment of the family A polymerase from *T. aquaticus* (Taq-Pol) and three variants of the family B polymerase from *P. furiosus* (Pfu-Pol): wild type, a 3′–5′ proofreading exonuclease-deficient mutant (D215A/E143A) [16], and an even more error-prone variant (D215A/E143A/D473G) [17]. In each case, the polymerase was used to fill gapped pSJ2 and pSJ3 in vitro, and successful extension was confirmed by EcoRI digestion and gel electrophoresis (Fig. 3). The filled plasmids were used for the transformation of *E. coli,* and the numbers of blue and white colonies were counted, enabling the determination of the mutation frequency (*MF*) (Table 4). To determine the error rate (*ER*) the following equation, which makes explicit the reasons for determining the detectable sites (*D*) and expression frequency (*P*), was used [12,22]:

$$\mathrm{ER}=\frac{N_{i}/N\times \mathrm{MF}}{D\times P}$$

where

*N_i_ *= number of a particular type of mutation (usually a deletion/insertion or base substitution)
*N *= total number of mutations
*MF *= observed mutation frequency – background mutation frequency
*D *= number of detectable sites for a particular mutation
*P *= probability of expressing the mutant *lacZα* gene (expression frequency).

With the above equation, the type of mutation (*N_i_*) can be determined only by DNA sequencing of mutant (white colonies) *lacZα* genes. In the absence of sequencing, *N_i_*/*N *= 1, and the equation can determine only total mutations [12,13,22].

The fidelity of wild-type Pfu-Pol has been characterized previously, and typically an error rate of between 1.3 × 10^−6^ and 1.6 × 10^−6^ is observed [6,8], similar to the values observed using both pSJ2 and pSJ3 (Table 4). Pfu-Pol lacking the 3′–5′ exonuclease (D215A/E143A) has an increased error rate due to loss of proofreading activity, and an approximately 2- to 4-fold decrease in fidelity is seen with the plasmid systems. An additional mutation (D473G) to a loop in the fingers domain further increases the number of mistakes made on replication [17], and an approximately 3-fold increase in error rate is observed when compared with Pfu-Pol (D215A/E143A) (equivalent to an ∼ 10-fold increase compared with the wild type). Finally, the fidelity of Taq-Pol, a family A DNA polymerase that lacks proofreading exonuclease activity, was determined. The accuracy of Taq-Pol is strongly dependent on the reaction conditions, and error rates of between 2 × 10^–4^ and 8 × 10^–6^ have been reported [6,8,13,21], in agreement with the value of 1 × 10^–5^ found with pSJ2 and pSJ3. One study [6] showed that Taq-Pol was approximately 6 times less accurate than Pfu-Pol, not too dissimilar to the decrease in fidelity found using pSJ2 and pSJ3 (Table 4). The good general agreement found between earlier investigations and the current studies with pSJ2 and pSJ3 suggests that both plasmids are suitable for determining the error rates of DNA polymerase.

The mutant (white) colonies from the pSJ2 assays of Pfu-Pol B wild type and the exonuclease-deficient variant were sequenced to determine further information on the mutation spectra. As was observed previously, a significant proportion of the mutants were C → T/G → A transitions, probably as a result of template strand cytosine deamination as described above for background mutation rate. This form of DNA damage was also observed in the pSJ1 and M13 methods, most likely also as a result of damage during substrate preparation [4,12,14]. However, because the background mutation frequency observed using pSJ2 and pSJ3 is lower, the influence of background mutations is reduced. To obtain rigorous data concerning the mutation spectrum of Pfu-Pol, a far higher number of mutants would obviously need to be sequenced. Nevertheless, this small data set confirms that each time a white mutant colony is observed with pSJ2, it is faithfully reflected in a DNA sequence error.

## Discussion

A considerably improved plasmid-based DNA polymerase fidelity assay has been described. The new plasmids, pSJ2 and pSJ3, are elaborations of an earlier version, pSJ1 [14], and contain a *lacZα* gene flanked by two nicking endonuclease sites. With pSJ2 and pSJ3, both the number of detectable sites and the expression frequency have been determined, factors necessary for determining polymerase error rates and not previously available for pSJ1. The expression frequency of 44.4% found for pSJ2 is near the theoretical maximum of 50% expected for random repair of mismatches, suggesting high plasmid integrity with minimal base damage. The critical step in applying pSJ2 and pSJ3 is gapping. As found with pSJ1, gapping cannot simply be carried out using heat to remove the strand excised in the nicking step. Rather, a complementary single-stranded competitor needs to be added to sequester the excised strand. The assay described in this article improves the preparation of relatively long competitors, using PCR followed by λ-exonuclease digestion, by exploiting the exonuclease resistance of phosphorothioate-protected primers. Although the phosphorothioate improvement consistently resulted in higher yields of competitor, PCR-based methods tend to give relatively small quantities of amplified material. Higher amounts of single-stranded DNA are available by direct chemical synthesis, but here the lengths that can be prepared become limiting. Thus, two competitors were required to successfully gap pSJ3; unfortunately, the use of four or five competitors to gap the longer pSJ2 failed. Gapping remains the most demanding part of the protocol, and research is still required to enable the production of long competitors in high quantity or to develop an alternative protocol. The use of BND–cellulose to isolate gapped plasmids represents a major improvement over gel-based purification techniques. Greater amounts can be prepared in a very straightforward manner and (importantly) the resulting gapped plasmids appear to be of much higher quality with little damage to the bases. This is evidenced by the very low background mutation rates seen for both pSJ2 and pSJ3 prepared using BND–cellulose, reduced approximately 5-fold as compared with gel-purified material. The low backgrounds seen with gapped pSJ2 (1 × 10^−4^) and pSJ3 (3 × 10^−5^) are extremely advantageous when studying high-fidelity polymerases. The suitability of the new plasmids was confirmed by determining the error rates of Pfu-Pol and Taq-Pol, which gave values similar to those obtained by a number of other methods. So far, pSJ2 and pSJ3 have been used only to test the fidelity of DNA polymerases in vitro. However, due to the compatibility of plasmids with many bacteria and eukaryotes, the in vivo study of DNA replication and repair should be possible, as has already been described for a plasmid-based mismatch repair activity assay [15].

## Figures and Tables

**Table 1:** Detectable sites within the *lacZα* gene.

*lacZα* gene source	Length of *lacZα* gene analyzed (bases)^a^	Number of detectable base substitutions	Number of detectable insertions and/or deletions	Total number of detectable sites
M13mp2^b^	278	241	199	440
pSJ2^c^	288	242	206	448
pSJ3^c^	163	166	163	329

a The sequences of the *lacZα* genes analyzed are given in Fig. 1A.

b Taken from work reported by Kunkel and coworkers [12,22].

c It has been assumed that the bases shared between pSJ2/pSJ3 and M13mp2 have the same “detectability”. The properties of the extra bases present in pSJ2 were determined by site-directed mutagenesis.

**Table 2:** Expression frequency of *lacZα* gene in pSJ2.

Nicked plasmid	Total number of colonies^a^	Number of mutant (white) colonies	% Mutant colonies	Expression frequency of *lacZα* gene
Heteroduplex (G:T mismatch)	7603	3379	44.4	0.444
Homoduplex (G:C base pair)	7393	0	0	Not applicable

a Obtained by summing three independent determinations.

**Table 3:** Background mutation frequencies of pSJ2 and pSJ3.

Plasmid	Total number of colonies	Number of mutant (white) colonies^a^	Mutation rate (%)	Mutation frequency^b^
pSJ2^c^	39,119	1	0.0026	2.6 × 10^−5^
pSJ2 (gapped, BND–cellulose purified)^c^	28,406	3	0.011	1.1 × 10^−4^
pSJ2 (gapped, gel purified)^c^	26,313	14	0.053	5.3 × 10^−4^
pSJ3 (gapped, BND–cellulose purified)^c^	32,519	1	0.0031	3.1 × 10^−5^
pSJ1^d^	–	–	0.08	8.0 × 10^−4^
M13mp2 (phage)^e^	–	–	0.04–0.06	4.0–6.0 × 10^−4^

a Plasmid DNA isolated from the four mutant colonies found with gapped pSJ2 and gapped pSJ3 (purified using BND–cellulose) was sequenced. Three of the mutations in the *lacZα* gene were C → T/G → A transitions. The fourth was a T → C/A → G transition.

b Number of errors made per base.

c Each data set with pSJ2 and pSJ3 was the sum of five independent experiments carried out in triplicate.

d Data taken from a previous study [14].

e Data taken from previous studies [12,22].

**Table 4:** Error rates of DNA polymerases determined using pSJ2 and pSJ3.

Polymerase	Gapped plasmid^a^	Number of colonies^b^	Number of mutant (white) colonies	Mutation frequency^c^	Error rate^d^
Pfu-Pol B	pSJ2	25,700	11	3.2 × 10^−4^	1.6 × 10^−6^
	pSJ3	20,116	11	5.2 × 10^−4^	3.5 × 10^−6^
Pfu-Pol B (D215A/E143A)^e^	pSJ2	14,601	20	1.3 × 10^−3^	6.3 × 10^−6^
	pSJ3	24,766	25	1.0 × 10^−3^	6.7 × 10^−6^
Pfu-Pol B (D215A/E143A/D473G) f	pSJ2	78,431	296	3.7 × 10^−3^	1.8 × 10^−5^
	pSJ3	38,836	141	3.6 × 10^−3^	2.4 × 10^−5^
Taq-Pol	pSJ2	46,239	98	2.0 × 10^−3^	1.0 × 10^−5^
	pSJ3	20,756	34	1.6 × 10^−3^	1.1 × 10^−5^

a All of the gapped plasmids used in these experiments had the coding strand (inner strand in Fig. 1B) removed by treatment with Nt.BbvC1 (pSJ2) or Nt.Bpu10I (pSJ3).

b Sum of three independent experiments, each consisting of five repeats.

c The mutation frequencies given here have had the background mutation frequencies found for gapped pSJ2 and pSJ3 subtracted.

d Error rates were calculated using the formula given in the text. An expression frequency (*P*) of 0.444 was used. In the absence of extensive DNA sequencing, an *N_i_*/*N* value of 1 was used and the number of detectable sites (*D*) was the sum of the values for base substitutions plus insertions/deletions, that is, 448 for pSJ2 and 329 for pSJ3 (Table 1).

e The mutation D215A/E143A disables the 3′–5′ proofreading exonuclease activity [16].

f The triple mutation D215A/E143A/D473G has even lower fidelity than the exo^–^ double mutant D215A/E143A [17].

## Footnotes

1 *Abbreviations used:* PCR, polymerase chain reaction; dNTP, deoxynucleotide triphosphate; BND–cellulose, benzoylated–naphthoylated DEAE–cellulose; Pfu-Pol B, *P. furiosus* family B DNA polymerase; Taq-Pol, *T. aquaticus* DNA polymerase.
