# Supplementary material for: A plasmid-based lacZα gene assay for DNA polymerase fidelity measurement
Source: Anal Biochem. 2013 Feb 15;433(2):153–61. doi: 10.1016/j.ab.2012.10.019 (PMC3552156; doi:10.1016/j.ab.2012.10.019)
Supplement: Supplementary data 1 — Supplementary material. [file mmc1.doc]

**SUPPLEMENTARY MATERIAL**

**Supplementary Figure 1. Key oligdoexynucleotides**

A) Oligodeoxynucleotides used to prepare competitor for gapping pSJ2 by the PCR/exonuclease III method.

| pSJ2 forward primer 5′- phosphorothioate (coding strand removal) | 5′-GsGsCsTGCGCAGCTGTTGGGAAG-3′ |
| --- | --- |
| pSJ2 reverse primer 5′-phosphate (coding strand removal) | 5′-pTCAGCGCAACGCAATTAATGTGAGTTAG-3′ |
| pSJ2 forward primer 5′-phosphate (non-coding strand removal) | 5′-ptgaggctgcgcagctgttgggaag-3′ |
| pSJ2 reverse primer 5′-phosphorothioate (non-coding strand removal) | 5′-GsCsGsCAACGCAATTAATGTGAGTTAG-3′ |

(p indicates a 5′-phosphate; s indicates a phosphorothioate)

B) Chemically synthesised oligodeoxynucleotides used for gapping of pSJ3 and attempted gapping of pSJ2.

| pSJ3 competitor 1 | 5′-AAGTTGGGTAACGCCAGGGTTTTCCCAGTCACGACGTTGTAAAAC  GACGGCCAGTGAATTCGTAATCATGGTCATAGCT-3′ |
| --- | --- |
| pSJ3 competitor 2 | 5′-GAACTGTTGGGAAGGGCGGTCGGTGCGGGCCTCTTCGCTATTACG  CCAGCTGGCGAAAGGGGGATGTGCTGCAAGGCGAT-3′ |
| pSJ2 competitor 1 | 5′-TGCGCAGCTGTTGGGAAGGGCGGTCGGTGCGGGCCTCTTCGCTAT  TACG-3′ |
| pSJ2 competitor 2 | 5′-CTGGCGAAAGGGGGATGTGCTGCAAGGCGATTAAGTTGGGTAAC  GCCAGGGTTTTCCCAGTC-3′ |
| pSJ2 competitor 3 | 5′-ACGTTGTAAAACGACGGCCAGTGAATTCGTAATCATGGTCATAGC  TGTTTCCTGTG-3′ |
| pSJ2 competitor 4 | 5′-ATTGTTATCCGCTCACAATTCCACACAACATACGAGCCGGAAGCA  TAAAGTGTAAAG-3′ |
| pSJ2 competitor 5 | 5′-TGGGGTGCCTAATGAGTGAGCTAACTCACATTAATTGCGTTGCGC  TG-3′ |
| pSJ2 competitor A | 5′-CAACATACGAGCCGGAAGCATAAAGTGTAAAGCCTGGGGTGCCT  AATGAGTGAGCTAACTCACATTAATTGCGTTGCG-3′ |
| pSJ2 competitor B | 5′-AAAACGACGGCCAGTGAATTCGTAATCATGGTCATAGCTGTTTCC  TGTGTGAAATTGTTATCCGCTCACAATTCC-3′ |
| pSJ2 competitor C | 5′-GCTGGCGAAAGGGGGATGTGCTGCAAGGCGATTAAGTTGGGTAA  CGCCAGGGTTTTCCCAGTCACGACG-3′ |
| pSJ2 competitor D | 5′-TGCGCAGCTGTTGGGAAGGGCGGTCGGTGCGGGCCTCTTCGCTAT  TACG |

pSJ3 competitors 1 and 2, in combination, were used to successfully remove the coding strand from pSJ3. Two sets of competitors (pSJ2 competitors 1-5 and A-D) were separately used in attempts to gap pSJ2. However, both sets gave negative results (see supplementary information, figure S3).

**Supplementary Figure 2. Detectable sites in pSJ2 and pSJ3.**

The continuous sequence (beginning with the red bases TCA and ending with the red bases CGCAGCC is the *lacZa* gene in pSJ2 used for fidelity determination. This is almost identical to the *lacZa* gene in M13mp2 (remaining sequence in black). Therefore, the “detectability” of the bases (i.e. if a change results in an inactive lacZa peptide and white colonies) in pSJ2 can be deduced from the wealth of data collected by Kunkel and co-workers for M13mp2 [12, 22]. Above the main sequence are base substitutions that give a detectable phenotype. Underlined positions in the promoter region give a detectable phenotype for insertions and deletions. The translation start site (ATG codon) is shown; all bases in the coding region are detectable following insertion or deletion. The pSJ2 bases shown in red are not present in M13mp2 but only one gives a detectable substitution phenotype (the A towards the 3'-end, on changing to C). pSJ3 is a truncated version of pSJ2 and the start and end positions are shown in green, as are all bases in pSJ3 that differ from those in pSJ2. None of these changes gave rise to a detectable substitution phenotype. The dam site, originally present as GATC in M13mp2 is shown boxed. The changes introduced into pSJ2 and pSJ3 to delete this site were also not detectable.

**Supplementary Figure 3. Attempted gapping of pSJ2 with five chemically synthesised oligodeoxynucleotides.**

Gel electrophoretic analysis of the generation of gapped pSJ2. After treatment of pSJ2 with Nb.BbvCI, the nicked (open-circular) form results, which can be converted to the linear form by treatment with EcoRI (see lanes marked 0 competitor). Heat/cool cycles with 5 synthetic oligodeoxynucleotides, each 50 - 80 bases long (excess used indicated above the gel lanes) (see supplementary material, figure S1 for sequences; these are the set pSJ2 competitors 1-5) produces little of the desired gapped plasmid. The nicked starting material and desired gapped product are poorly resolved. However, EcoRI converts the nicked plasmid (but not the gapped) to the well separated linear form, enabling analysis of the progress of the gapping reactions. The yield of the gapped product is represented by the ratios of the nicked/gapped and linear bands seen after EcoRI digestion, higher intensity in the former band indicating more product. As can be seen most of the material runs as the linear form after EcoRI treatment, indicating it remains as the starting nicked plasmid. This figure should be compared with figure 2A in the main paper. Gapping with a long single competitor was much more successful. Using the set pSJ2 competitors A-D (supplementary material, figure S1) gave a similar result.

**Supplementary Figure 4. Preparation and analysis of a nicked heteroduplex plasmid for expression frequency determination.**

A) Scheme outlining the preparation of a heteroduplex plasmid containing a single nick. An initial round of site directed mutagenesis on pSJ2 removes the ucs, giving pSJ2A. A second round introduces a stop codon into the *lacZa* gene by changing a single C to T on the coding strand (the inner strand in al the above plasmids). This gives a *lacZαS* (S = stop) gene in pSJ2B. pSJ2A is subsequently converted to a single-stranded circle and pSJ2B to a linear duplex, as illustrated. Mixing and heating these two products yields the desired heteroduplex plasmid with a *lacZαH* (H = heteroduplex) gene, containing a G:T mismatch and a single nick. A suitable control was generated by using pSJ2A to prepare the linear duplex DNA. B) Analysis of the steps shown in scheme A by agarose gel electrophoresis.
